# NO Scavenging through Reductive Nitrosylation of Ferric *Mycobacterium tuberculosis* and *Homo sapiens* Nitrobindins

**DOI:** 10.3390/ijms21249395

**Published:** 2020-12-10

**Authors:** Giovanna De Simone, Alessandra di Masi, Chiara Ciaccio, Massimo Coletta, Paolo Ascenzi

**Affiliations:** 1 Department of Sciences, Roma Tre University, Viale Guglielmo Marconi 446, 00146 Roma, Italy; giovanna.desimone@uniroma3.it (G.D.S.); alessandra.dimasi@uniroma3.it (A.d.M.); 2 Department of Clinical Sciences and Translational Medicine, University of Roma “Tor Vergata”, Via Montpellier 1, I-00133 Roma, Italy; chiara.ciaccio@uniroma2.it (C.C.); coletta@seneca.uniroma2.it (M.C.); 3 Interdepartmental Laboratory of Electron Microscopy, Roma Tre University, Via della Vasca Navale 79, I-00146 Roma, Italy

**Keywords:** nitrobindin, heme-protein, NO scavenging, reductive nitrosylation

## Abstract

Ferric nitrobindins (Nbs) selectively bind NO and catalyze the conversion of peroxynitrite to nitrate. In this study, we show that NO scavenging occurs through the reductive nitrosylation of ferric *Mycobacterium tuberculosis* and *Homo sapiens* nitrobindins (*Mt*-Nb(III) and *Hs*-Nb(III), respectively). The conversion of *Mt*-Nb(III) and *Hs*-Nb(III) to *Mt*-Nb(II)-NO and *Hs*-Nb(II)-NO, respectively, is a monophasic process, suggesting that over the explored NO concentration range (between 2.5 × 10^−5^ and 1.0 × 10^−3^ M), NO binding is lost in the mixing time (i.e., ^NO^*k*_on_ ≥ 1.0 × 10^6^ M^−1^ s^−1^). The pseudo-first-order rate constant for the reductive nitrosylation of *Mt*-Nb(III) and *Hs*-Nb(III) (i.e., *k*) is not linearly dependent on the NO concentration but tends to level off, with a rate-limiting step (i.e., *k*_lim_) whose values increase linearly with [OH^−^]. This indicates that the conversion of *Mt*-Nb(III) and *Hs*-Nb(III) to *Mt*-Nb(II)-NO and *Hs*-Nb(II)-NO, respectively, is limited by the OH^−^-based catalysis. From the dependence of *k*_lim_ on [OH^−^], the values of the second-order rate constant *k*_OH−_ for the reductive nitrosylation of *Mt*-Nb(III)-NO and *Hs*-Nb(III)-NO were obtained (4.9 (±0.5) × 10^3^ M^−1^ s^−1^ and 6.9 (±0.8) × 10^3^ M^−1^ s^−1^, respectively). This process leads to the inactivation of two NO molecules: one being converted to HNO_2_ and another being tightly bound to the ferrous heme-Fe(II) atom.

## 1. Introduction

Globins are globular ferrous heme-proteins that have evolved across all domains of life to sense, bind, store, and transport diatomic gases, as well as to catalyze the synthesis and scavenging of reactive nitrogen and oxygen species [1,2,3]. They are composed of six or four α-helical segments, which are shaped around heme with a 3/3- or 2/2-fold. In 3/3 globins, the A, B, and E α-helices face the heme distal side and the F, G, and H α-helices are in front of the proximal side. In 2/2 globins, two antiparallel pairs of α-helices B/E and G/H encompass the heme [2,3,4,5,6].

In contrast, nitrophorins (NPs) and nitrobindins (Nbs) are the only all-β-barrel heme-proteins displaying a ferric heme-Fe atom [7,8,9,10,11,12,13]. The eight-stranded β-barrel NPs have been described only in the hematophagous bug *Rhodnius prolixus* (*Rp*-NP), which stores NO in its salivary glands. When the insect feeds on its victim, its saliva is pumped into the victim’s tissues and NO is released, with consequent vasodilation. Then, *Rp*-NPs bind the host’s histamine, reducing inflammation and itch [14,15,16,17,18,19]. In contrast, NPs from the bedbug *Cimex lectularius* display a mixed α-helical/β-barrel-fold, highlighting the remarkable evolution of proteins that assist insects in blood feeding [20].

The physiological role(s) of the evolutionary conserved 10-stranded β-barrel Nbs is still uncertain [10,11,12,13]. Ferric *Arabidopsis thaliana* Nb has been hypothesized to transport and to release NO at the infection site after wounding and pathogenic infections; then, NO may reduce O_2_ to the superoxide radical, increasing pathogen burden [10]. Moreover, ferric *Mycobacterium tuberculosis* and *Homo sapiens* Nbs (*Mt*-Nb(III) and *Hs*-Nb(III), respectively) have been reported to selectively bind NO and to catalyze peroxynitrite scavenging, protecting both organisms from peroxynitrite-mediated nitration [13,21].

Unlike monomeric NPs and bacterial and plant Nbs [7,8,9,10,13,15,16,18], *Hs*-Nb corresponds to the *C*-terminal domain of the single-chain nuclear peroxynitrite isomerase THAP4. Interestingly, it could act as a NO sensor modulating THAP4 transcriptional function, residing at the *N*-terminal modified zinc finger domain [13].

To shed light on the NO chemistry of Nbs, the reductive nitrosylation of *Mt*-Nb(III) and *Hs*-Nb(III) has been investigated and analyzed in parallel with that of prototypical all-α-helical heme-proteins. Interestingly, the values of the dissociation equilibrium constant for NO binding to *Mt*-Nb(III) and *Hs*-Nb(III) (i.e., *K*) and of the second-order rate constant of the OH^−^-mediated conversion of *Mt*-Nb(III)-NO to *Mt*-Nb(II)-NO and of *Hs*-Nb(III)-NO to *Hs*-Nb(II)-NO (i.e., *k*_OH−_) are closely similar to those of all-α-helical heme-proteins. The much faster value of the second-order rate constant for *Mt*-Nb(III) and *Hs*-Nb(III) nitrosylation (i.e., ^NO^*k*_on_) is likely related to the much more open heme distal side. The present results highlight the inactivation of two NO molecules: one being converted to HNO_2_, and another being tightly bound to the heme-Fe(II) atom. In this respect, *Hs*-Nb may act as a NO/O_2_ sensor, modulating the transcriptional activity of THAP4.

## 2. Results

**Scheme 1:**
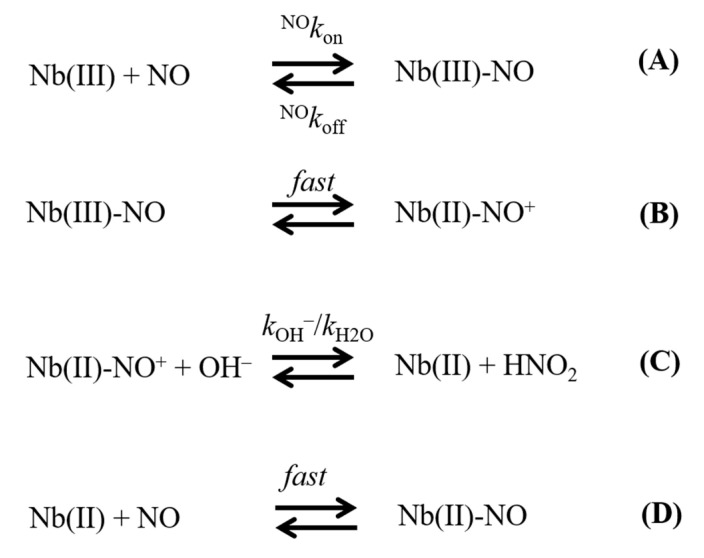
Mechanism of reductive nitrosylation of *Mt*-Nb(III) and *Hs*-Nb(III).

The reductive nitrosylation of ferric heme-proteins is an alkaline-driven process involving two NO molecules, one being converted to HNO_2_ and the other being trapped tightly to the ferrous heme-Fe atom (Scheme 1) [22,23,24,25,26,27,28,29].

At pH 7.0 and 22.0 °C, *Mt*-Nb(III) and *Hs*-Nb(III) reversibly bind NO [13], whereas between pH 7.8 and 9.2 (*T* = 20.0 °C), both Nbs undergo irreversible reductive nitrosylation (i.e., NO-dependent formation of Nb(II)-NO) (present study). In fact, *Mt*-Nb(II)-NO and *Hs*-Nb(II)-NO convert to *Mt*-Nb(II) and *Hs*-Nb(II) instead of *Mt*-Nb(III) and *Hs*-Nb(III) by pumping off NO.

**Figure 1:**
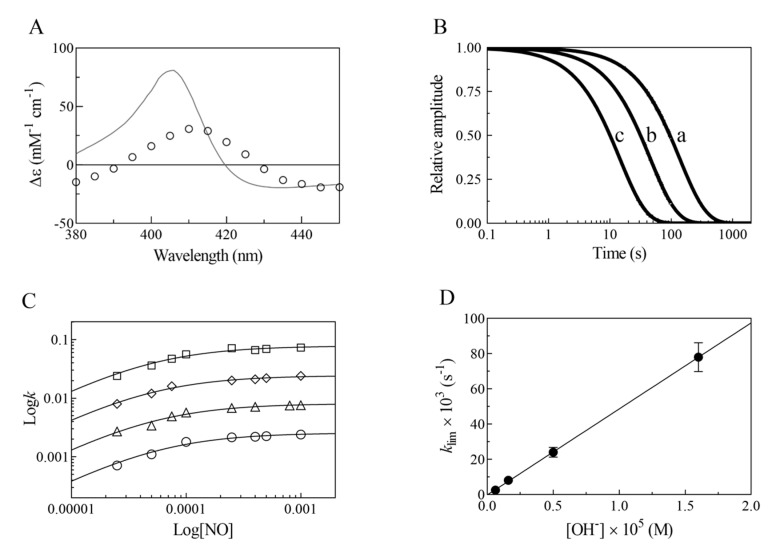
Reductive nitrosylation of *Mt*-Nb(III) at 20.0 °C. (**A**) Difference optical absorption spectrum of *Mt*-Nb(III) minus *Mt*-Nb(II)-NO (static difference optical absorption spectrum; continuous line) and of *Mt*-Nb(III)-NO minus *Mt*-Nb(II)-NO (kinetic difference optical absorption spectrum; circles) at pH 7.8. (**B**) Time course of reductive nitrosylation of *Mt*-Nb(III) at pH 8.2 (a), 8.7 (b), and 9.2 (c). Values of the rate constant *k* were calculated according to Equation (1): 7.3 × 10^−3^ s^−1^ (a), 2.2 × 10^−2^ s^−1^ (b), and 6.9 × 10^−2^ s^−1^ (c). The *Mt*-Nb(III) concentration was 4.1 × 10^−6^ M. The NO concentration was 5.0 × 10^−4^ M. (**C**) Dependence of Log*k* (s^−1^) on Log[NO] (M) at pH 7.8 (circles), 8.2 (triangles), 8.7 (diamonds), and 9.2 (squares). The continuous lines were calculated according to Equation (3) with the following parameters: pH 7.8: *K* = 5.7 (±0.6) × 10^−5^ M and *k*_lim_ = 2.5 (±0.3) × 10^−3^ s^−1^; pH 8.2: *K* = 5.2 (±0.6) × 10^−5^ M and *k*_lim_ = 8.1 (±0.9) × 10^−3^ s^−1^; pH 8.7: *K* = 4.8 (±0.5) × 10^−5^ M and *k*_lim_ = 2.4 (±0.3) × 10^−2^ s^−1^; pH 9.2: *K* = 5.0 (±0.6) × 10^−5^ M and *k*_lim_ = 7.8 (±0.9) × 10^−2^ s^−1^. (**D**) Dependence of *k*_lim_ on the OH^−^ concentration. The continuous line was calculated according to Equation (4) with *k*_OH−_ = 4.9 (±0.5) × 10^3^ M^−1^ s^−1^. Where not shown, the error bars were smaller than the symbol.

**Figure 2:**
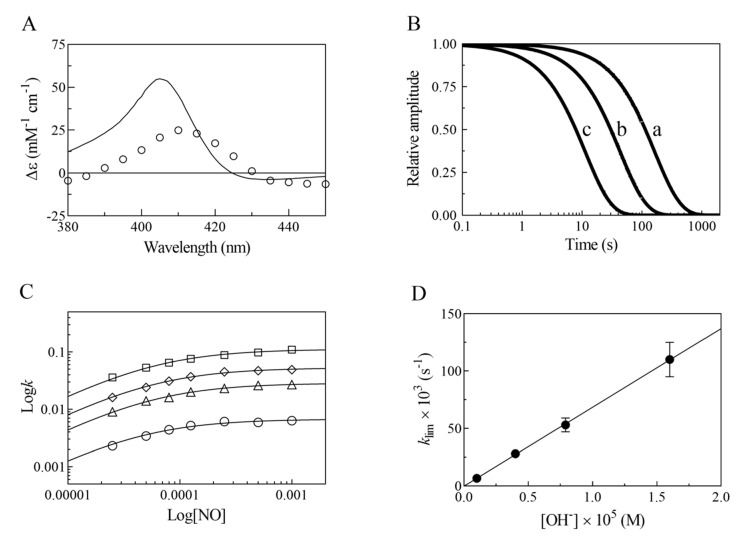
Reductive nitrosylation of *Hs*-Nb(III) at 20.0 °C. (**A**) Difference optical absorption spectrum of *Hs*-Nb(III) minus *Hs*-Nb(II)-NO (static difference optical absorption spectrum; continuous line) and of *Hs*-Nb(III)-NO minus *Hs*-Nb(II)-NO (kinetic difference optical absorption spectrum; circles) at pH 8.0. (**B**) Time course of reductive nitrosylation of *Hs*-Nb(III) at pH 8.0 (a), 8.6 (b), and 9.2 (c). Values of the rate constant *k* were calculated according to Equation (1): 6.1 × 10^−3^ s^−1^ (a), 2.3 × 10^−2^ s^−1^ (b), and 8.8 × 10^−2^ s^−1^ (c). The *Hs*-Nb(III) concentration was 3.5 × 10^−6^ M. The NO concentration was 5.0 × 10^−4^ M. (**C**) Dependence of Log*k* (s^−1^) on Log[NO] (M) at pH 8.0 (circles), 8.6 (triangles), 8.9 (diamonds), and 9.2 (squares). The continuous lines were calculated according to Equation (3) with the following parameters: pH 8.0: *K* = 4.4 (±0.5) × 10^−5^ M and *k*_lim_ = (6.7 (±0.7) × 10^−3^ s^−1^; pH 8.6: *K* = 5.6 (±0.7) × 10^−5^ M and *k*_lim_ = 2.8 (±0.3) ×10^−2^ s^−1^; pH 8.9: *K* = 5.6 (±0.7) × 10^−5^ M and *k*_lim_ = 5.3 (±0.6) × 10^−2^ s^−1^; pH 9.2: *K* = 5.7 (±0.6) × 10^−5^ M and *k*_lim_ = 1.1 (±0.2) × 10^−1^ s^−1^. (**D**) Dependence of *k*_lim_ on the OH^−^ concentration. The continuous line was calculated according to Equation (4) with *k*_OH−_ = 6.9 (±0.7) × 10^3^ M^−1^ s^−1^. Where not shown, the error bars were smaller than the symbol.

Upon mixing, *Mt*-Nb(III) and *Hs*-Nb(III) with the NO solutions, the absorbance changes of *Mt*-Nb(III) and *Hs*-Nb(III) nitrosylation (i.e., *Mt*-Nb(III)-NO and *Hs*-Nb(III)-NO formation; Scheme 1A) are likely lost in the dead-time of the rapid-mixing stopped-flow apparatus (~2 ms), as expected over this NO concentration range (spanning between 2.5 × 10^−5^ and 1.0 × 10^−3^ M) on the basis of the reported ^NO^*k*_on_ values [13] (Table 1). As a matter of fact, immediately after mixing (i.e., within the first 10 ms), the absorbance spectra are already those corresponding to *Mt*-Nb(III)-NO and *Hs*-Nb(III)-NO. Then, the absorbance spectra of *Mt*-Nb(III)-NO and *Hs*-Nb(III)-NO gradually change to those of *Mt*-Nb(II)-NO and *Hs*-Nb(II)-NO. Accordingly, the static difference absorbance spectra of *Mt*-Nb(III) minus *Mt*-Nb(II)-NO and *Hs*-Nb(III) minus *Hs*-Nb(II)-NO, obtained by keeping the *Mt*-Nb(III) and *Hs*-Nb(III) solutions under NO at *p* = 760.0 mm Hg, differ from the difference absorbance spectra obtained after rapid-mixing the *Mt*-Nb(III) and *Hs*-Nb(III) solutions with the NO solutions, since in the last case they correspond to those of *Mt*-Nb(III)-NO minus *Mt*-Nb(II)-NO and *Hs*-Nb(III)-NO minus *Hs*-Nb(II)-NO (Figure 1A and Figure 2A, respectively). This confirms that the observed time courses (Figure 1B and Figure 2B, respectively) reflect the conversion of *Mt*-Nb(III)-NO to *Mt*-Nb(II)-NO and of *Hs*-Nb(III)-NO to *Hs*-Nb(II)-NO.

The time-courses of *Mt*-Nb(III) and *Hs*-Nb(III) reductive nitrosylation are monophasic for 94 ± 6% of their course (Figure 1B and Figure 2B, respectively), corresponding to Scheme 1B–D. The values of the pseudo-first-order rate constant for *Mt*-Nb(III) and *Hs*-Nb(III) reductive nitrosylation (i.e., *k*) do not increase linearly with the NO concentration but tend to level off (Figure 1C and Figure 2C, respectively), highlighting the occurrence of a fast pre-equilibrium process followed by a rate-limiting step. The analysis of data shown in Figure 1C and Figure 2C according to Equation (3) allowed us to determine the values of the dissociation equilibrium constant for *Mt*-Nb(III) and *Hs*-Nb(III) nitrosylation (i.e., *K*), and of the first-order rate constant limiting the reductive nitrosylation process (i.e., *k*_lim_). The values of *K* are pH-independent (ranging between 4.4 (±0.5) × 10^−5^ and 5.7 (±0.6) × 10^−5^ M) (Table 1) and agree with those previously obtained at pH 7.0 and 22.0 °C for the reversible nitrosylation of *Mt*-Nb(III) and *Hs*-Nb(III) (i.e., 5.0 (±1.5) × 10^−5^ and 4.4 (±1.2) × 10^−5^ M) [13].

The values of *k*_lim_ for the reductive nitrosylation of *Mt*-Nb(III) and *Hs*-Nb(III) increase linearly with pH (Figure 1D and Figure 2D, respectively). The analysis of data according to Equation (4) allowed us to determine the values of *k*_OH−_ for the OH^−^-catalyzed conversion of *Mt*-Nb(II)-NO^+^ to *Mt*-Nb(II) and *Hs*-Nb(II)-NO^+^ to *Hs*-Nb(II), respectively, from the slope of the linear plots of *k*_lim_ versus [OH^−^] (Table 1). The intercept of the straight lines with the *y* axis (i.e., *k*_H2O_) is close to 0 s^−1^, indicating that OH^−^ catalyzes the reductive nitrosylation of *Mt*-Nb(III) and *Hs*-Nb(III) more efficiently than H_2_O.

## 3. Discussion

Nitrobindins constitute a new class of heme-proteins, characterized by a peculiar all-β-barrel structural arrangement [10,11,12,13]. Thus far, they have been found in *M. tuberculosis*, *A. thaliana*, and *H. sapiens*, where they are a *C*-terminal domain of the single-chain nuclear protein THAP4. Since the heme iron is stably in the Fe(III) form, their possible physiological role has been proposed to be NO sensing [10,11,13]; therefore, the characterization of the interaction of *Mt*-Nb(III) and *Hs*-Nb(III) with NO and of their pseudo-enzymatic role as NO scavengers is of the utmost importance. Of note, *Mt*-Nb(III) and *Hs*-Nb(III) react quickly with NO, with the values of the second-order rate constant ^NO^*k*_on_ being 1.8 × 10^6^ M^−1^ s^−1^ and 1.1 × 10^6^ M^−1^ s^−1^, respectively (Table 1); then, the Fe(III)-NO complex undergoes a slow reductive process, bringing about the formation of HNO_2_ and Fe(II)-NO through a series of pH-dependent reactions (Scheme 1).

The data reported here allow us to draw the following conclusions:

(i) The very fast NO binding kinetics of Mt-Nb(III) and Hs-Nb(III) [13], so as to be lost in the dead-time of the rapid-mixing stopped-flow apparatus at the employed NO concentrations (ranging between 2.5 × 10^−5^ and 1.0 × 10^−3^ M), reflect a specific feature of Nbs, which is shared with NPs [9,31], since all other heme-proteins show an about 10-fold slower reactivity (Table 1).
(ii) Despite the very different structural organization of the heme-proteins examined (all-β-barrel versus all-α-helix structures) [4,5,6,11,13,32,33,34,35,36], the values of K for NO binding to ferric heme-proteins are closely similar (ranging between 1.4 × 10^−5^ and 2.1 × 10^−4^ M) (present study and [13,22,23,24,25,26,27,30]) (Table 1). This suggests a balance between the NO association and dissociation rate constants among most heme-proteins, such that the energy barriers for ligand entry and exit are affected to the same extent by structural constraints.
(iii) The linear dependence of k on [OH^–^] indicates that no additional features are involved in the irreversible reductive nitrosylation of *Mt*-Nb(III) and *Hs*-Nb(III). Moreover, the values of k_OH–_ for ferric heme-protein reductive nitrosylation range between 1.7 × 10^2^ M^−1^ s^−1^ and 6.9 × 10^3^ M^−1^ s^−1^ [13,22,23,24,25,26,27,30] (Table 1), possibly reflecting the different anion accessibility to the heme pocket and/or the heme-Fe(III) protein reduction potentials [6,22,23,34,36,37,38,39]. The close similarity of k_OH-_ between all-β-barrel Nbs and all-α-helical Hb indicates that the grossly different folding [4,6,10,11,13,36,40,41] does not significantly affect anion accessibility to the heme pocket.
(iv) The rate of the conversion of heme-Fe(II)-NO^+^ to heme-Fe(II) + HNO_2_ (Scheme 1C) represents the rate-limiting step of heme-protein reductive nitrosylation (present study and [22,23,24,25,26,27,28,30]). Of note, the OH^–^ species catalyzes the conversion of Mt-Nb(II)-NO^+^ to Mt-Nb(II) + HNO_2_ and of Hs-Nb(II)-NO^+^ to Hs-Nb(II) + HNO_2_ much more efficiently than H_2_O (k_H2O_ ~ 0 s^−1^). This may reflect either the higher affinity of negatively charged ligands for ferric heme-proteins with respect to uncharged compounds and/or the deprotonation rate of the incoming ligand [5,37,42].
(v) NO binding to ferrous heme-proteins (Scheme 1D) is known to also be very fast in Nbs [10]; therefore, it does not represent the rate-limiting step of the reductive nitrosylation process.

## 4. Materials

*Hs*-Nb and *Mt*-Nb were cloned, expressed, and purified as described previously [13]. The *Hs*-Nb and *Mt*-Nb concentration was determined spectrophotometrically using the following extinction coefficients at λ_max_ = 407 nm: 100 and 147 mM cm^−1^, respectively [13].

Gaseous NO (from Linde Caracciolossigeno S.r.l., Roma, Italy) was purified by flowing through a glass column packed with NaOH pellets, and then by passage through a trapping solution containing 20 mL of 5.0 M NaOH to remove traces of impurities; the NO pressure was 760.0 mmHg [43]. The stock NO solution was prepared anaerobically by keeping the degassed 2.0 × 10^−3^ M bis-tris propane buffer solution (pH 7.0) under NO in a closed vessel at *P* = 760.0 mm Hg (*T* = 20.0 °C). The solubility of NO in the aqueous buffered solution is 2.05 × 10^−3^ M, at *P* = 760.0 mm Hg and *T* = 20.0 °C [44]. The concentration of NO in the solution was determined, under anaerobic conditions and in the absence of the gaseous phase, by titration of ferrous horse heart Mb monitored by visible absorption spectroscopy [45].

All the other chemicals were obtained from Merck KGaA (Darmstadt, Germany). All products were of analytical grade and used without purification unless stated.

All the experiments were carried out with the SFM-20/MOS-200 rapid-mixing stopped-flow apparatus (BioLogic Science Instruments, Claix, France); the dead-time of the stopped-flow apparatus was ~2 ms and the observation chamber was 1 cm.

## 5. Methods

The reductive nitrosylation of *Mt*-Nb(III) and *Hs*-Nb(III) was investigated between pH 7.8 and 9.2 (5.0 × 10^–2^ M bis-tris propane buffer), at *T* = 20.0 °C. The kinetics of the reductive nitrosylation of *Mt*-Nb(III) and *Hs*-Nb(III) were analyzed in the framework of the minimum reaction mechanism (Scheme 1) [22,23,24,25,26,27,28,29].

It is noteworthy that Scheme 1 indeed represents a redox enzymatic process, where NO scavenging is associated with the reduction of the heme-Fe(III) and nitrogen oxidation from NO and HNO_2_; thus, NO is the substrate and HNO_2_ is the product, with the whole chemical transformation being rate-limited by the OH^−^-dependent rate-limiting step *k*_OH−_.

The values of the apparent pseudo-first-order rate constant for the reductive nitrosylation (i.e., *k*) were obtained by rapid-mixing the *Mt*-Nb(III) and *Hs*-Nb(III) solution (final concentration ranging between 3.5 × 10^−6^ and 4.1 × 10^−6^ M) with the NO solution (final concentration ranging between 2.5 × 10^−5^ and 1.0 × 10^−3^ M) under anaerobic conditions. No gaseous phase was present. The reductive nitrosylation of *Mt*-Nb(III) was monitored by single-wavelength stopped-flow spectroscopy between 380 and 450 nm. The amplitude of the time courses was normalized at 407 nm. The values of *k* were obtained according to Equation (1) [22,23,24,25,26,27,28,29]:

[*Mt*-Nb(III)]_t_ = [*Mt*-Nb(III)]_i_ × e ^−*k* × *t*^ (1)

The values of the equilibrium constant *K* (corresponding to ^NO^*k*_off_/^NO^*k*_on_ referring to Scheme 1A) and of the rate-limiting pseudo-first-order rate constant *k*_lim_ (referring to Scheme 1B–D) were obtained from the dependence of *k* on the NO concentration (i.e., [NO]), ranging between 2.5 × 10^−5^ and 1.0 × 10^−3^ M, according to Equations (2) and (3) [22,23,24,25,26,27,28,29]:

*k* = (*k*_lim_ × ^NO^*k*_on_ × [NO])/(^NO^*k*_off_ + ^NO^*k*_on_ × [NO]) (2)

which, considering that ^NO^*k*_off_ = *K* × ^NO^*k*_on_, can be also expressed as:

*k* = (*k*_lim_ × [NO])/(*K* + [NO]) (3)

The values of the apparent second-order (i.e., *k*_OH−_) and of the first-order (i.e., *k*_H2O_) rate constants for the OH^−^- and H_2_O-catalyzed conversion of *Mt*-Nb(III)-NO^+^ to *Mt*-Nb(II), respectively, (Scheme 1C) were determined from the dependence of *k*_lim_ on the OH^−^ concentration (i.e., [OH^−^]) according to Equation (4) [22,23,24,25,26,27,28,29]:

*k* = *k*_OH−_ × [OH^−^] + *k*_H2O_ (4)

## 6. Conclusions

The present results highlight the role of *Mt*-Nb(III) and *Hs*-Nb(III) as NO scavengers through the reductive nitrosylation reaction, which leads to the inactivation of two NO molecules. In spite of their much higher reactivity with NO to form the *Mt*-Nb(III)-NO and *Hs*-Nb(III)-NO species, respectively, the rate-limiting step for the conversion of Fe(II)-NO^+^ to Fe(II) and HNO_2_ is essentially similar to that of all-α-helical heme-proteins, resulting only slightly faster than what was observed for human Hb, *Methanosarcina acetivorans* Pgb, and *Glycine max* legHb (Table 1). This indicates that the structural determinants controlling these reactions are different. Indeed, while the very different NO reactivity reflects the diverse structural organization of the heme distal pocket of all-α-helical and all-β-barrel heme-proteins, the similar reactivity of OH^−^ is controlled by still undetermined factors. Reflecting the NO/O_2_ ratio, the reductive nitrosylation of *Mt*-Nb(III) and *Hs*-Nb(III) occurs at high NO concentration, whereas the oxidation of *Mt*-Nb(II)-NO and *Hs*-Nb(II)-NO takes place at high O_2_ levels. In this context, *Hs*-Nb may act as a NO/O_2_ sensor, modulating the transcriptional activity of THAP4.

## Figures and Tables

**Table 1:** Values of ^NO^*k*_on_, *K*, and *k*_OH−_ for the reductive nitrosylation of ferric heme-proteins.

Heme-Protein	^NO^*k*_on_ (M^−1^ s^−1^)	*K* (M)	*k*_OH−_ (M^−1^ s^−1^)
*Mt*-Nb	1.8 × 10^6 a^	5.2 × 10^−5 b^	4.9 × 10^3 c^
*Hs*-Nb	1.1 × 10^6 a^	5.3 × 10^−5 d^	6.9 × 10^3 c^
*Mt*-trHbN	1.4 × 10^5 e^	1.4 × 10^−5 e^	1.7 × 10^2 f^
*Mt*-trHbO	9.2 × 10^3 e^	1.5 × 10^−5 e^	2.4 × 10^2 f^
*Cj-*trHbP	9.8 × 10^4 e^	5.4 × 10^−5 e^	9.1 × 10^2 f^
*Methanosarcina acetivorans* Pgb	4.8 × 10^4 g^	6.1 × 10^−5 g^	2.9 × 10^3 h^
*Glycine max* legHb	1.4 × 10^5 i^	2.1 × 10^−5 i^	3.3 × 10^3 j^
Horse heart Mb	6.8 × 10^4 k^	1.2 × 10^−4 k^	3.9 × 10^2 l^
Sperm whale Mb	1.9 × 10^5 m^	7.7 × 10^−5 m^	3.2 × 10^2 n^
Human Hb	--- ^o^	8.3 × 10^−5 p^	3.2 × 10^3 p^
Human Hb:Hp1-1	--- ^o^	9.1 × 10^−5 q^	4.9 × 10^3 r^
Human Hb:Hp2-2	--- ^o^	1.7 × 10^−4 q^	6.7 × 10^3 r^

^a^ Value of ^NO^*k*_on_ obtained at pH 7.0 and *T* = 20 °C. From [13]. ^b^ Average value, values of *K* were obtained between pH 7.8 and 9.2 at *T* = 20.0 °C. Present study. ^c^ *T* = 20.0 °C. Present study. ^d^ Average value, values of *K* were obtained between pH 8.0 and 9.2 at *T* = 20.0 °C. Present study. ^e^ Average value, values of ^NO^*k*_on_ and *K* were obtained between pH 8.4 and 9.4 at *T* = 20.0 °C. From [27]. ^f^ *T* = 20.0 °C. From [27]. ^g^ pH 7.2 and 22.0 °C. From [26]. ^h^ *T* = 22.0 °C. From [26]. ^i^ pH 7.3 and *T* = 20.0 °C. From [24]. ^j^ Room temperature. From [24]. ^k^ pH 9.2 and *T* = 20.0 °C. From [30]. ^l^ *T* = 20.0 °C. From [30]. ^m^ pH 8.79 and room temperature. From [23]. ^n^ Room temperature. From [23]. ^o^ Kinetics of NO binding to human Hb(III), human Hp1-1:Hb(III), and human Hp2-2:Hb(III) are very fast and therefore they are lost in the mixing time [23,28]. ^p^ Room temperature. From [23]. ^q^ pH 8.2 and *T* = 20 °C. From [28]. ^r^ *T* = 20 °C. [28].
